# Emotional Dysregulation and Stress-Related Psychopathology in Workers Exposed to Occupational Stress

**DOI:** 10.3390/bs16010105

**Published:** 2026-01-13

**Authors:** Antonello Veltri, Maria Francesca Beatino, Martina Corsi, Martina Chiumiento, Fabrizio Caldi, Giovanni Guglielmi, Rudy Foddis, Giulio Perugi, Rodolfo Buselli

**Affiliations:** 1 Center for Work-Related Stress and Occupational Mental Disorders, Azienda Ospedaliero-Universitaria Pisana, 56124 Pisa, Italy; 2 Occupational Health Unit, Azienda Ospedaliero-Universitaria Pisana, 56124 Pisa, Italy; 3 Department of Mental Health and Addictions, Azienda USL Toscana Nord Ovest, 56121 Pisa, Italy; 4 Department of Translational Research and of New Surgical and Medical Technologies, University of Pisa, 56126 Pisa, Italy; 5 Psychiatry Unit, Department of Clinical and Experimental Medicine, University of Pisa, 56126 Pisa, Italy

**Keywords:** emotional dysregulation, RIPoSt-40, stress, work, depression, adjustment disorders

## Abstract

Emotional dysregulation (ED) reflects a heightened reactivity to stimuli, characterized by excessive negative affect and impulsive behaviors. This study aimed to evaluate ED in workers seeking care for occupational stress and to examine its associations with sociodemographic characteristics, occupational stress, and the severity of anxiety and depressive symptoms. Eighty-seven workers referred for work-related stress were assessed using the Psychological Stress Measure (PSM) and the Job Content Questionnaire (JCQ) for stress, the Beck Depression Inventory-II (BDI-II) and the Self-Rating Anxiety Scale (SAS) for psychopathology, and the RIPoSt-40 for ED. Group comparisons and correlation analyses were conducted using parametric or non-parametric tests, as appropriate. Forty-six percent of participants met criteria for Adjustment Disorders and 54% for Major Depressive Disorder. No significant differences between diagnostic groups emerged for ED or symptom severity. Women reported higher perceived stress and anxiety than men. Negative ED domains—affective instability, negative emotionality, and emotional impulsivity—showed moderate-to-strong positive correlations with stress, anxiety, and depressive symptoms. Affective instability was also related to job stress dimensions, correlating negatively with decision latitude and positively with job demands. Negative emotional dysregulation appears to be a transdiagnostic vulnerability factor for stress-related psychopathology. Screening for ED may support early detection and targeted preventive interventions in occupational settings.

## 1. Introduction

Prolonged exposure to occupational stressors represents a major risk factor for the emergence of depressive, anxiety, and burnout syndromes, as well as for other stress-related mental disorders in vulnerable individuals (Buselli et al., 2016, 2022). The detrimental effects of chronic work stress are mediated not only by psychological mechanisms but also by biological pathways, influencing neuroplasticity and contributing to the epigenetic modulation of genes crucial for brain function (Buselli et al., 2019, 2023; Veltri et al., 2024, 2025a). In this context, a closer integration between occupational medicine and psychiatry is increasingly recognized as essential for the prevention of stress-induced psychiatric conditions (Buselli et al., 2020; Veltri et al., 2025b). Nevertheless, the current literature still lacks sufficient evidence regarding vulnerability and resilience factors to work-related stress—information that would be of great value for occupational physicians aiming to identify workers who are more fragile and at higher risk of developing psychopathology in response to chronic occupational strain.

Recent findings indicate that personality traits play a crucial role in determining individual variability in stress reactivity (Xin et al., 2017). Likewise, the subjective intensity of perceived occupational stress is influenced by affective temperament profiles (Alarcon et al., 2009; Tei-Tominaga et al., 2012; Buselli et al., 2022). Studies conducted in Japan have shown that depressive and anxious temperaments are associated with greater sensitivity to job-related stressors, heightened stress perception, and an increased risk of depression, whereas a hyperthymic temperament appears to exert a protective effect (Sakai et al., 2005; Tei-Tominaga et al., 2012; Deguchi et al., 2016). Moreover, Italian research has highlighted that workers with a family history of psychiatric disorders, pathological personality traits, or lifetime mood spectrum symptoms—especially manic-type—are at increased risk of developing severe psychopathology when exposed to occupational stress (Buselli et al., 2016).

The inability to effectively modulate emotional states, commonly referred to as emotional dysregulation (ED), represents a construct of major importance in both psychiatric research and clinical practice. Drawing on existing literature on ED assessment tools, Marwaha et al. (2014) proposed a conceptualization comprising three main components: affective lability, emotional intensity, and regulatory capacity. Accordingly, ED can be broadly described as “rapid shifts of intense emotional states, accompanied by difficulty in managing these fluctuations or their behavioral outcomes.” It reflects an increased tendency to respond to stimuli with excessive negative affect and impulsive behaviors compared to individuals of similar age or developmental stage. Clinically, ED is recognized as a transdiagnostic feature observable across multiple psychiatric conditions—including ADHD, borderline personality disorder, and mood spectrum disorders—rather than being confined to any specific diagnostic category (De Prisco et al., 2023). Moreover, it has been identified as a robust predictor of suicidal behavior (Rigucci et al., 2021).

Based on the current literature, the present study aimed to clarify the role of emotional dysregulation (ED) as a specific vulnerability factor within the framework of occupational mental health. The primary objective was to assess the levels of ED in a clinical sample of workers referred for secondary-level evaluation due to psychological distress related to occupational stress. Furthermore, the investigation sought to explore the associations between ED domains, sociodemographic characteristics, perceived occupational stress levels, and the severity of anxiety and depressive symptoms. In line with these objectives, several research hypotheses were formulated. First, it was hypothesized that higher levels of negative emotional dysregulation (comprising affective instability, negative emotionality, and emotional impulsivity) would be positively associated with greater perceived occupational stress and increased psychiatric symptom severity. Second, we hypothesized that ED would act as a transdiagnostic feature, manifesting with comparable levels across different stress-related clinical diagnoses.

## 2. Materials and Methods

### 2.1. Subjects

This study examined a cohort of 87 workers experiencing psychological discomfort related to occupational stress, who were consecutively enrolled from January to December 2024 at the Center for Work-Related Stress and Occupational Mental Disorders of the Azienda Ospedaliero-Universitaria Pisana. All participants were referred to the Center for secondary-level evaluation by occupational physicians from either their companies or public occupational health services. The Center is part of a comprehensive research program investigating the effects of occupational stress on mental health. Workers reporting high levels of work-related stress are referred for evaluation and undergo an integrated, multidisciplinary assessment aimed at identifying potential psychiatric conditions and clarifying their etiological relationship with occupational stressors. The present study represents a specific arm of this broader research project, focusing on emotional dysregulation (ED) as a potential vulnerability factor in response to work-related stress.

Participants were excluded if they were younger than 18 or older than 65 years; if their current psychopathological symptoms or recurrent mood episodes had emerged prior to exposure to stressful work situations; if they had insufficient proficiency in Italian to complete self-report questionnaires; or if they were unable to provide informed consent.

The study protocol was approved by the Ethics Committee of the Area Vasta Nord Ovest of Tuscany, in accordance with the Declaration of Helsinki (World Medical Association, 2013). All participants provided written informed consent before enrollment.

### 2.2. Clinical Assessment

All enrolled participants underwent an integrated assessment process including a detailed medical examination performed by an occupational physician, followed by psychiatric and psychological evaluations. Afterward, each participant completed a battery of self-report questionnaires and rating scales, described below.

Sociodemographic and occupational data were gathered using a structured self-report form that collected information on job category, employment type, duration of service in current and previous positions, and details regarding the organization’s size and industrial sector.

To establish psychiatric diagnoses and exclude any history of recurrent depressive episodes or other lifetime psychiatric disorders preceding exposure to occupational stress, all participants were interviewed using the Structured Clinical Interview for DSM-5—Clinician Version (SCID-5-CV) (First & Williams, 2016), in conjunction with the psychiatric evaluation.

Work-related stress was evaluated through the Italian version of the Job Content Questionnaire (JCQ), a widely adopted self-report tool assessing the psychosocial features of the work environment (Karasek et al., 1998; Baldasseroni et al., 2001). The JCQ comprises 49 Likert-scale items ranging from “strongly disagree” to “strongly agree,” and investigates three core domains: (a) decision latitude, (b) psychological job demands, and (c) social support, in line with the high-demand/low-control/low-support job strain model. Specifically, the decision latitude subscale measures the degree of autonomy and control over work activities, integrating both decision authority and skill discretion. The psychological job demands subscale captures the cognitive and emotional effort required by job tasks, while the social support subscale assesses perceived assistance and empathy from supervisors and coworkers, emphasizing the interpersonal dimension of the workplace (Karasek et al., 1998).

Perceived psychological stress was assessed using the Psychological Stress Measure (PSM), a validated 49-item self-report questionnaire developed from stress-related descriptors derived through focus groups (Lemyre & Tessier, 1988; Di Nuovo et al., 2000).

Emotional dysregulation was evaluated with the Reactivity, Intensity, Polarity, and Stability questionnaire (RIPoSt-40) (Brancati et al., 2019). The 40 items, rated on a six-point Likert scale from 1 (“never”) to 6 (“always”), yield four primary subscales—affective instability (AI), positive emotionality (PE), negative emotionality (NE), and emotional impulsivity (EI)—as well as a second-order negative emotional dysregulation (NED) factor derived from AI, NE, and EI. This scoring rationale follows the psychometric structure validated by Brancati et al. (2019), where NED emerged as a robust global indicator of the maladaptive affective reactivity typically observed in stress-related and mood disorders. By using the NED factor, we were able to capture the overall severity of the negative dysregulation spectrum, while the primary subscales provided more granular detail on specific emotional manifestations.

Depressive symptom severity was measured using the Italian version of the Beck Depression Inventory-II (BDI-II), a well-validated 21-item self-report scale (Beck et al., 1996; Montano & Flebus, 2006). The BDI-II includes cognitive-affective and somatic subcomponents, showing Cronbach’s α values of 0.86 and 0.65, respectively.

Anxiety levels were assessed with the Italian version of the Self-Rating Anxiety Scale (SAS) (Zung, 1971; Conti, 1999), a 20-item self-report instrument designed to quantify state anxiety. The SAS demonstrates strong internal consistency (Cronbach’s α = 0.86), with raw scores ranging from 20 to 80 and recommended thresholds of 40 for clinical evaluation and 36 for screening purposes (Dunstan & Scott, 2020).

### 2.3. Statistical Analysis

All data were entered into a dedicated database and analyzed using MedCalc software (version 12.7). The distribution of continuous variables was examined with the Kolmogorov–Smirnov test, which indicated that all variables, except the RIPoSt-40 AI and NED scores, followed a normal distribution. Variables with a normal distribution are reported as mean ± standard deviation (SD), whereas non-normally distributed variables are presented as median and interquartile range [IQR]. Categorical data are summarized as absolute frequencies and percentages. Comparisons between groups were conducted using independent-samples *t*-tests for normally distributed variables and Mann–Whitney U tests for those not meeting normality assumptions. Differences in categorical variables were assessed using the Chi-square test. Correlations between continuous variables were evaluated using Pearson’s correlation coefficient for normally distributed data, and Spearman’s rank correlation coefficient for non-normally distributed measures. Given the exploratory nature of this study, no formal adjustments for multiple comparisons were applied to the correlation analyses. This approach was chosen to maintain adequate sensitivity in identifying potential associations within this clinical population, minimizing the risk of Type II errors, while prioritizing the interpretation of consistent patterns of significance across the assessed domains. For all statistical analyses, a two-tailed *p*-value < 0.05 was considered statistically significant.

## 3. Results

### 3.1. Characteristics of Participants

Sociodemographic, occupational, and clinical characteristics of the participants, along with stress and rating scale scores, are summarized in Table 1.

The study sample comprised 87 workers, including 35 males (40.2%), with a mean age of 52.2 ± 8.6 years (SD). Most participants had at least 13 years of education (74.1%), were employed in the private sector (60%), and held stable positions (96.5%). According to DSM-5 diagnostic criteria (American Psychiatric Association, 2013), 46% of participants were diagnosed with Adjustment Disorder (AD), while 54% met criteria for Major Depressive Disorder (MDD).

No significant differences emerged between AD and MDD groups in terms of anxiety severity (SAS scores), depressive symptoms (BDI-II scores), perceived psychological stress (PSM scores), or occupational stress (JCQ subscale scores). Similarly, no significant differences were observed in any of the RIPoSt-40 subscale scores between the two groups.

Male and female participants did not differ significantly with respect to age, educational level, employment stability, depressive symptom severity, occupational stress, or emotional dysregulation levels. However, female participants exhibited significantly higher scores on both the PSM (*p* < 0.001) and SAS (*p* = 0.01) compared with males.

### 3.2. Correlation Analyses

Results of the correlation analyses among clinical variables are presented in Table 2.

Significant associations were found between RIPoSt-40 subscale scores and measures of stress and psychopathology. All negative domains of the RIPoSt-40—emotional impulsivity (EI), affective instability (AI), negative emotionality (NE), and the composite Negative Emotional Dysregulation (NED) score—showed moderate to strong positive correlations with PSM, BDI-II, and SAS scores (for *r* and *p* values see Table 2).

Among occupational stress dimensions, only the RIPoSt-40 AI subscale showed significant correlations: it was negatively correlated with the JCQ Decision Latitude score (*r* = −0.23, *p* = 0.05) and positively correlated with the JCQ Job Demands score (*r* = 0.24, *p* = 0.04). In contrast, the PE (positive emotionality) subscale did not show significant correlations with any of the variables examined.

## 4. Discussion

The present study explored emotional dysregulation (ED) as a potential vulnerability factor among workers experiencing psychological discomfort related to occupational stress. Our findings indicate that ED—particularly its negative dimensions of affective instability (AI), negative emotionality (NE), and emotional impulsivity (EI)—is significantly associated with perceived psychological stress, depressive symptoms, and anxiety severity. These results support the hypothesis that difficulties in regulating emotions may amplify psychological reactivity to work-related stressors.

Consistent with previous literature, our findings further confirm that ED is a transdiagnostic construct relevant across different psychiatric conditions. No significant differences in ED levels were observed between participants diagnosed with Adjustment Disorder (AD) and those with Major Depressive Disorder (MDD), suggesting that emotional dysregulation functions as a general vulnerability factor rather than being specific to a single diagnostic category. This is in line with earlier evidence showing that ED constitutes a core dimension underlying mood spectrum and stress-related disorders (De Prisco et al., 2023; Rigucci et al., 2021). In addition to comparable ED levels, the AD and MDD groups did not differ in perceived psychological and occupational stress, anxiety, or depressive symptoms. This finding resonates with previous observations highlighting the diagnostic overlap between MDD and AD, particularly in mild to moderate MDD (Casey et al., 2006). In the present study, the absence of differences may reflect the nature of the depressive episodes considered—namely, first-onset MDD episodes temporally linked to psychosocial stressors. The phenomenology of such episodes often closely mirrors that of Adjustment Disorder, consistent with the “kindling” hypothesis (Post, 1992), which posits that psychosocial stressors play a dominant role in triggering initial depressive episodes, whereas later episodes become increasingly autonomous from external stress. In this context, our cohort may reflect what is referred to as “reactive depression” (Showraki, 2019), where the episode is closely linked to identifiable life stressors. This may explain the overlapping emotional and clinical features observed in our sample.

Gender-related patterns also emerged: female participants reported significantly higher levels of perceived psychological stress and anxiety, while no differences were observed in depression severity, occupational stress, or ED. This pattern mirrors previous findings suggesting greater affective reactivity and stress perception among women, likely reflecting both biological and psychosocial mechanisms influencing stress responsivity (Rohleder et al., 2001; Sakai et al., 2005; Schmaus et al., 2008).

Correlation analyses confirmed a robust association between the negative domains of ED (AI, NE, EI, and NED composite) and psychological distress measures. These findings underscore the close interplay between impaired emotional regulation and increased affective reactivity under occupational stress. Prior studies have shown that emotion dysregulation predicts both depressive and anxiety symptomatology and mediates the impact of stress exposure (Freudenthaler et al., 2017; Arpacı & Tanrıverdi, 2024). Longitudinal research further suggests a bidirectional mechanism, whereby emotional dysregulation exacerbates affective symptoms, which in turn reinforce regulatory deficits—forming a self-perpetuating cycle of vulnerability to stress-related psychopathology (Chan et al., 2023). Notably, AI also correlated with objective job stress parameters: negatively with decision latitude and positively with job demands, as measured by the Job Content Questionnaire. In other words, greater mood variability and reactivity is associated with experiencing higher job pressure and lower decision-making autonomy with less control over tasks, conditions known to increase occupational stress. AI has been conceptualized as rapid and intense affective shifts with delayed recovery following psychosocial triggers (Koenigsberg, 2010). Previous studies indicate that high job demands combined with low decision latitude predict greater depressive and anxiety symptoms (Paterniti et al., 2002). Moreover, temperament traits linked to mood instability (e.g., cyclothymic temperament) have been associated with higher perceived job stress and burnout, particularly under low-control conditions (Jaracz et al., 2017). Experimental evidence similarly shows that reduced decision latitude amplifies negative affect during high-demand tasks (Hutt & Weidner, 1993). Collectively, these findings suggest that individuals with high AI may be particularly vulnerable to both the structural and emotional demands of the workplace. It could be hypothesized that, mechanistically, AI may function as a cognitive-emotional filter that distorts the perception of workplace stressors; individuals with high AI may experience occupational tasks as more overwhelming (perceived demand) and feel less capable of managing them (perceived control), regardless of the objective workload. However, a bidirectional influence cannot be ruled out: an objectively high-strain environment may act as a persistent trigger that destabilizes emotional regulation in vulnerable workers, creating a maladaptive feedback loop. Conversely, positive emotionality (PE) was not significantly associated with stress or psychopathology, suggesting that positive affect alone may be insufficient to counterbalance the impact of negative emotional dysregulation.

Overall, our results highlight the relevance of ED in the assessment of occupational stress. Workers exhibiting pronounced emotional dysregulation may face increased risk of developing clinically significant psychopathology under chronic work stress. From an occupational health perspective, screening for ED and implementing targeted interventions—such as emotion regulation training, stress management programs, or cognitive-behavioral strategies—could enhance resilience and prevent progression toward more severe psychiatric outcomes.

The present study has several limitations that should be acknowledged. First, its cross-sectional design does not allow causal inferences regarding the relationships between emotional dysregulation (ED), stress perception, and psychopathology. Although ED is generally conceptualized as a stable, trait-like construct, it can nonetheless be influenced by acute affective states. Therefore, elevated ED scores might partly reflect the participants’ current emotional distress rather than an enduring vulnerability factor. Longitudinal or prospective designs would be necessary to clarify whether ED predicts the onset or persistence of stress-related disorders over time. Second, the modest sample size (n = 87) may have resulted in limited statistical power to detect smaller effect sizes or differences between diagnostic subgroups. Specifically, the lack of significant differences in emotional dysregulation levels between workers with Adjustment Disorder and those with Major Depressive Disorder should be interpreted with caution, as it may be influenced by the study’s limited capacity to identify subtle variations between these diagnostic categories. Larger cohorts are necessary to confirm whether these findings reflect a true transdiagnostic overlap or are a consequence of the sample constraints. Moreover, the sample consisted primarily of middle-aged workers with stable employment who were referred for evaluation within an occupational health framework. This homogeneity enhances internal consistency but restricts the generalizability of the findings to younger workers, individuals in precarious or high-turnover jobs. Third, all variables were assessed using self-report questionnaires, which, while widely used and validated, may be subject to reporting biases, including social desirability and mood-congruent recall effects. Such biases may lead to over- or underestimation of both emotional dysregulation and perceived stress.

## 5. Conclusions

In conclusion, this study demonstrates that negative emotional dysregulation, specifically its facets of affective instability, negative emotionality, and emotional impulsivity, is robustly associated with perceived psychological stress, depressive symptoms, and anxiety severity in workers. Our findings confirm that emotional dysregulation functions as a transdiagnostic vulnerability factor, as no significant differences in ED levels were observed between participants with different stress-related psychiatric disorders.

Based on these results, we postulate that impaired emotional regulation may amplify an individual’s psychological reactivity to occupational stressors, potentially fostering a self-perpetuating cycle of vulnerability to stress-related psychopathology. From a preventive perspective, we suggest that incorporating ED assessment into routine occupational health surveillance could represent a critical step for the early identification of at-risk workers. We further hypothesize that targeted interventions, such as emotion regulation training and stress management programs, could enhance workforce resilience and prevent the progression of reactive psychological distress into more severe psychiatric conditions. Given the moderate-to-strong correlations observed between negative ED domains and clinical symptoms, future research should focus on replicating these findings in larger cohorts across different occupational sectors to confirm the robustness of ED as a transdiagnostic vulnerability marker. Furthermore, longitudinal research is required to definitively establish the causal role of ED in the onset of occupational mental disorders and to investigate its biological underpinnings, its interaction with temperament and personality, and the effectiveness of interventions aimed at improving emotion regulation skills in workplace contexts.

## Figures and Tables

**Table 1:** Sociodemographic, occupational, and clinical characteristics of the participants.

	Participants (n = 87)
Age (mean ± SD)	52.2 ± 8.6
Gender M (%)	40.2
Education ≥ 13 years (%)	74.1
Public company workers (%)	40.0
Private company workers (%)	60.0
Temporary job (%)	3.5
Stable job (%)	96.5
MDD diagnosis	54.0
AD diagnosis	46.0
JCQ Decision Latitude (mean ± SD)	60.2 ± 14.3
JCQ Job Demands (mean ± SD)	37.0 ± 6.1
JCQ Social Support (mean ± SD)	15.0 ± 4.1
BDI-II total score (mean ± SD)	23.0 ± 12.6
SAS total score (mean ± SD)	45.8 ± 10.6
PSM total score (mean ± SD)	115.6 ± 24.2
RIPoSt-40 AI score (median [IQR])	26.0 [18.5–34.5]
RIPoSt-40 PE score (mean ± SD)	35.3 ± 12.0
RIPoSt-40 NE score (mean ± SD)	30.9 ± 11.1
RIPoSt-40 EI score (mean ± SD)	19.5 ± 7.5
RIPoSt-40 NED score (median [IQR])	75.0 [58.5–91.5]

Legend—MDD: Major Depressive Disorder; AD: Adjustment Disorders, JCQ: Job Content Questionnaire; BDI-II: Beck Depression Inventory-II; SAS: Self-rating Anxiety Scale; PSM: Psychological Stress Measure, RIPoSt-40: Reactivity, Intensity, Polarity and Stability Questionnaire (AI: Affective Instability, PE: Positive Emotionality, NE: Negative Emotionality, EI: Emotional Impulsivity, NED: Negative Emotional Dysregulation).

**Table 2:** Correlation coefficients (*r*) between RIPoSt-40 subscale scores, stress levels, and psychopathological symptoms severity.

Variables	JCQ Decision Latitude Score	JCQ Job Demands Score	JCQ Social Support Score	PSM Score	BDI-II Score	SAS Score
RIPoSt-40 AI score (Spearman)	**−0.23** ***p* = 0.05**	**0.24** ***p* = 0.04**	−0.10 *p* = 0.41	**0.44** ***p* < 0.001**	**0.49** ***p* = 0.0001**	**0.30** ***p* = 0.01**
RIPoSt-40 PE score (Pearson)	0.17 *p* = 0.15	0.13 *p* = 0.29	−0.07 *p* = 0.55	0.17 0.17	0.05 0.68	0.05 0.71
RIPoSt-40 NE score (Pearson)	−0.21 *p* = 0.08	0.11 *p* = 0.38	−0.11 *p* = 0.37	**0.62** ***p* < 0.0001**	**0.67** ***p* < 0.0001**	**0.57** ***p* < 0.0001**
RIPoSt-40 EI score (Pearson)	−0.05 *p* = 0.68	0.05 *p* = 0.66	0.03 *p* = 0.81	**0.50** ***p* < 0.0001**	**0.46** ***p* < 0.001**	**0.31** ***p* = 0.01**
RIPoSt-40 NED score (Spearman)	−0.17 *p* = 0.16	0.14 *p* = 0.24	−0.08 *p* = 0.51	**0.56** ***p* < 0.0001**	**0.62** ***p* < 0.0001**	**0.41** ***p* = 0.001**

Legend—JCQ: Job Content Questionnaire; BDI-II: Beck Depression Inventory-II; SAS: Self-rating Anxiety Scale; PSM: Psychological Stress Measure, RIPoSt-40: Reactivity, Intensity, Polarity and Stability Questionnaire (AI: Affective Instability, PE: Positive Emotionality, NE: Negative Emotionality, EI: Emotional Impulsivity, NED: Negative Emotional Dysregulation). Values in bold indicate statistically significant results (*p* < 0.05).

## Data Availability

The original contributions presented in this study are included in the article. Further inquiries can be directed to the corresponding author.

## References

[B1-behavsci-16-00105] Alarcon G., Eschleman K. J., Bowling N. A. (2009). Relationships between personality variables and burnout: A meta-analysis. Work & Stress.

[B2-behavsci-16-00105] American Psychiatric Association (2013). Diagnostic and statistical manual of mental disorders.

[B3-behavsci-16-00105] Arpacı R., Tanrıverdi D. (2024). The mediating role of emotion regulation difficulties in the correlation between mindfulness and psychological resilience in patients diagnosed with depression. The Journal of Nervous and Mental Disease.

[B4-behavsci-16-00105] Baldasseroni A., Camerino D., Cenni P., Cesana G., Fattorini E., Ferrario M., Mariani M., Tartaglia R. (2001). La valutazione dei fattori psicosociali. Proposta della versione italiana del job content questionnaire di R.A. Karasek [Assessment of psychosocial factors. Proposal of the Italian version of R.A. Karasek’s job content questionnaire]. ISPESL.

[B5-behavsci-16-00105] Beck A. T., Steer R. A., Brown G. K. (1996). Manual for the beck depression inventory second edition (BDI-II).

[B6-behavsci-16-00105] Brancati G. E., Barbuti M., Pallucchini A., Cotugno B., Schiavi E., Hantouche E. G., Perugi G. (2019). Reactivity, intensity, polarity and stability questionnaire (RIPoSt-40) assessing emotional dysregulation: Development, reliability and validity. Journal of Affective Disorders.

[B7-behavsci-16-00105] Buselli R., Del Guerra P., Caldi F., Veltri A., Battaglia S., Baldanzi S., Girardi M., Sallese D., Dell’Osso L., Cristaudo A. (2020). Mental disability management within occupational health surveillance. La Medicina del Lavoro.

[B8-behavsci-16-00105] Buselli R., Veltri A., Baldanzi S., Bozzi S., Marino R., Chiumiento M., Dell’Osso L., Cristaudo A. (2016). Work-related stress disorders: Variability in clinical expression and pitfalls in psychiatric diagnosis. La Medicina del Lavoro.

[B9-behavsci-16-00105] Buselli R., Veltri A., Baldanzi S., Marino R., Bonotti A., Chiumiento M., Girardi M., Pellegrini L., Guglielmi G., Dell’Osso L., Cristaudo A. (2019). Plasma brain-derived neurotrophic factor (BDNF) and serum cortisol levels in a sample of workers exposed to occupational stress and suffering from adjustment disorders. Brain and Behavior.

[B10-behavsci-16-00105] Buselli R., Veltri A., Corsi M., Marino R., Baldanzi S., Chiumiento M., Caldi F., Foddis R., Guglielmi G., Cristaudo A., Dell’Osso L., Carmassi C. (2022). Affective temperament and mood spectrum symptoms in workers suffering from work-related stress disorders. Journal of Affective Disorders.

[B11-behavsci-16-00105] Buselli R., Veltri A., Corsi M., Marino R., Necciari G., Baldanzi S., Chiumiento M., Del Lupo E., Foddis R., Caldi F., Kozakova M., Guglielmi G., Palombo C. (2023). Heart rate variability, serum cortisol levels and temperament in a sample of workers exposed to occupational stress: A preliminary report. Psychology, Health & Medicine.

[B12-behavsci-16-00105] Casey P., Maracy M., Kelly B. D., Lehtinen V., Ayuso-Mateos J.-L., Dalgard O. S., Dowrick C. (2006). Can adjustment disorder and depressive episode be distinguished? Results from ODIN. Journal of Affective Disorders.

[B13-behavsci-16-00105] Chan K. M. Y., Hong R. Y., Ong X. L., Cheung H. S. (2023). Emotion dysregulation and symptoms of anxiety and depression in early adolescence: Bidirectional longitudinal associations and the antecedent role of parent-child attachment. The British Journal of Developmental Psychology.

[B14-behavsci-16-00105] Conti L., Conti L. (1999). The Zung self-rating anxiety scale (SAS). Repertorio delle scale di valutazione in psichiatria.

[B15-behavsci-16-00105] De Prisco M., Oliva V., Fico G., Radua J., Grande I., Roberto N., Anmella G., Hidalgo-Mazzei D., Fornaro M., de Bartolomeis A., Serretti A., Vieta E., Murru A. (2023). Emotion dysregulation in bipolar disorder compared to other mental illnesses: A systematic review and meta-analysis. Psychological Medicine.

[B16-behavsci-16-00105] Deguchi Y., Iwasaki S., Konishi A., Ishimoto H., Ogawa K., Fukuda Y., Nitta T., Inoue K. (2016). The usefulness of assessing and identifying workers’ temperaments and their effects on occupational stress in the workplace. PLoS ONE.

[B17-behavsci-16-00105] Di Nuovo S., Rispoli L., Genta E. (2000). Misurare lo stress.

[B18-behavsci-16-00105] Dunstan D. A., Scott N. (2020). Norms for Zung’s self-rating anxiety scale. BMC Psychiatry.

[B19-behavsci-16-00105] First M. B., Williams J. B. W. (2016). SCID-5-CV: Structured clinical interview for DSM-5 disorders: Clinician version.

[B20-behavsci-16-00105] Freudenthaler L., Turba J. D., Tran U. S. (2017). Emotion regulation mediates the associations of mindfulness on symptoms of depression and anxiety in the general population. Mindfulness.

[B21-behavsci-16-00105] Hutt J., Weidner G. (1993). The effects of task demand and decision latitude on cardiovascular reactivity to stress. Behavioral Medicine.

[B22-behavsci-16-00105] Jaracz M., Rosiak I., Bertrand-Bucińska A., Jaskulski M., Nieżurawska J., Borkowska A. (2017). Affective temperament, job stress and professional burnout in nurses and civil servants. PLoS ONE.

[B23-behavsci-16-00105] Karasek R., Brisson C., Kawakami N., Houtman I., Bongers P., Amick B. (1998). The job content questionnaire (JCQ): An instrument for internationally comparative assessments of psychosocial job characteristics. Journal of Occupational Health Psychology.

[B24-behavsci-16-00105] Koenigsberg H. W. (2010). Affective instability: Toward an integration of neuroscience and psychological perspectives. Journal of Personality Disorders.

[B25-behavsci-16-00105] Lemyre L., Tessier R. (1988). Mesure de stress psychologique (MSP): Se sentir stressé-e [Measurement of psychological stress: To feel stressed]. Canadian Journal of Behavioural Science/Revue Canadienne des Sciences du Comportement.

[B26-behavsci-16-00105] Marwaha S., He Z., Broome M., Singh S. P., Scott J., Eyden J., Wolke D. (2014). How is affective instability defined and measured? A systematic review. Psychological Medicine.

[B27-behavsci-16-00105] Montano A., Flebus G. B. (2006). Presentazione del Beck depression inventory-seconda edizione (BDI-II): Conferma della sruttura bifattoriale in un campione di popolazion italiana [Presentation of the Beck depression inventory-second edition (BDI-II): Confirmation of the bifactorial structure in a sample of the Italian population]. Psicoterapia Cognitiva e Comportamentale.

[B28-behavsci-16-00105] Paterniti S., Niedhammer I., Lang T., Consoli S. M. (2002). Psychosocial factors at work, personality traits and depressive symptoms. Longitudinal results from the GAZEL study. The British Journal of Psychiatry: The Journal of Mental Science.

[B29-behavsci-16-00105] Post R. M. (1992). Transduction of psychosocial stress into the neurobiology of recurrent affective disorder. The American Journal of Psychiatry.

[B30-behavsci-16-00105] Rigucci S., Sarubbi S., Erbuto D., Rogante E., Hantouche E. G., Innamorati M., Lester D., Pompili M. (2021). Negative emotion dysregulation is linked to the intensity of suicidal ideation in a mixed inpatient sample. Journal of Affective Disorders.

[B31-behavsci-16-00105] Rohleder N., Schommer N. C., Hellhammer D. H., Engel R., Kirschbaum C. (2001). Sex differences in glucocorticoid sensitivity of proinflammatory cytokine production after psychosocial stress. Psychosomatic Medicine.

[B32-behavsci-16-00105] Sakai Y., Akiyama T., Miyake Y., Kawamura Y., Tsuda H., Kurabayashi L., Tominaga M., Noda T., Akiskal K., Akiskal H. (2005). Temperament and job stress in Japanese company employees. Journal of Affective Disorders.

[B33-behavsci-16-00105] Schmaus B. J., Laubmeier K. K., Boquiren V. M., Herzer M., Zakowski S. G. (2008). Gender and stress: Differential psychophysiological reactivity to stress reexposure in the laboratory. International Journal of Psychophysiology: Official Journal of the International Organization of Psychophysiology.

[B34-behavsci-16-00105] Showraki M. (2019). Reactive depression: Lost in translation!. The Journal of Nervous and Mental Disease.

[B35-behavsci-16-00105] Tei-Tominaga M., Akiyama T., Sakai Y. (2012). Effect of affective temperaments assessed by the TEMPS-A on the relationship between work-related stressors and depressive symptoms among workers in their twenties to forties in Japan. Depression Research and Treatment.

[B36-behavsci-16-00105] Veltri A., Caldi F., Buselli R. (2025b). Mental health and rapid societal evolution: An occupational prevention challenge. Occupational Medicine.

[B37-behavsci-16-00105] Veltri A., Nicolì V., Marino R., Rea F., Corsi M., Chiumiento M., Giangreco M., Caldi F., Guglielmi G., Foddis R., Coppedè F., Silvestri R., Buselli R. (2024). Plasma brain-derived neurotrophic factor (BDNF) levels and BDNF promoters’ DNA methylation in workers exposed to occupational stress and suffering from psychiatric disorders. Brain Sciences.

[B38-behavsci-16-00105] Veltri A., Nicolì V., Stoccoro A., Marino R., Rea F., Corsi M., Chiumiento M., Caldi F., Guglielmi G., Silvestri R., Foddis R., Coppedè F., Buselli R. (2025a). Occupational stress and epigenetic regulation: Methylation of the glucocorticoid receptor gene promoter in depressed workers. Stress.

[B39-behavsci-16-00105] World Medical Association (2013). World medical association declaration of Helsinki: Ethical principles for medical research involving human subjects. JAMA.

[B40-behavsci-16-00105] Xin Y., Wu J., Yao Z., Guan Q., Aleman A., Luo Y. (2017). The relationship between personality and the response to acute psychological stress. Scientific Reports.

[B41-behavsci-16-00105] Zung W. W. (1971). A rating instrument for anxiety disorders. Psychosomatics.

